# Understanding Service Utilization & Quality of Life Post-Hospice Live Discharge

**DOI:** 10.1093/geroni/igaf122.2620

**Published:** 2025-12-31

**Authors:** Stephanie P Wladkowski, Leslie Hinyard, Ruaa Al Juboori, Verna Hendricks-Ferguson, Cara Wallace

**Affiliations:** Bowling Green State University, Bowling Green, Ohio, United States; Saint Louis University School of Medicine, St. Louis, Missouri, United States; University of Mississippi, Oxford, Mississippi, United States; Trudy Busch Valentine School of Nursing, St. Louis, Missouri, United States; Saint Louis University, Saint Louis, Missouri, United States

## Abstract

Following a live discharge from hospice, patients and their caregivers often need to re-establish or transition to community resources as hospice services are no longer available. Although patients may no longer qualify for hospice, they often continue to experience complex medical and psychosocial needs. Therefore, coordinated service utilization following live discharge is critical to ensuring continuity of care, minimizing emergency room visits or caregiver burnout, and supporting quality of life (QOL) for both patients and caregivers. This study surveyed patient-caregiver dyads (n = 24) approximately 3 months following a live discharge due to extended prognosis, to better understand the services utilized and their QOL post-hospice care. Participants were an average age of 86 (SD 12.1), 83% female, and 79% White with 50% of participating living at home, while 42% were in long-term care. Within the first 3 months post discharge, 12.5% reported visiting an urgent care at least once, 8% reported at least one ER visit, and 8% reported at least one hospitalization. Further, 62% reported an established primary care provider while only 33% reported using home health, and less than half (46%) reported use of paid caregivers. Lastly, only 25% of patients reported “Good” QOL in the immediate post-discharge period. Results demonstrate that in the initial post-discharge period, medical service utilization is relatively low as is reported QOL. Research, clinical, and policy implications will be discussed.

